# Aggressiveness in Judokas and Team Athletes: Predictive Value of Personality Traits, Emotional Intelligence and Self-Efficacy

**DOI:** 10.3389/fpsyg.2021.824123

**Published:** 2022-01-10

**Authors:** Nemanja Stanković, Dušan Todorović, Nikola Milošević, Milica Mitrović, Nenad Stojiljković

**Affiliations:** ^1^Faculty of Sport and Physical Education, University of Niš, Niš, Serbia; ^2^Faculty of Philosophy, Department of Psychology, University of Niš, Niš, Serbia

**Keywords:** judo, team sports, HEXACO model of personality, self-efficacy, emotional intelligence

## Abstract

Combat sports and martial arts are often associated with aggressiveness among the general public, although data on judo and/or martial arts and aggressiveness seem to be unclear. This research aims to compare athletes who have trained judo for a prolonged time (minimum 5 years) and athletes from various team sports, primarily regarding the manifestation of aggression, but also regarding personality traits, emotional intelligence, and self-efficacy. Also, the potential predictive value of personality traits, emotional intelligence, and self-efficacy for aggression within subsamples of judokas and team athletes was tested. The research findings showed that professional judo athletes are characterized by a low degree of aggression, especially low indirect and physical manifestations of aggression. In addition, the personality traits Honesty-Humility and Openness to experience are well expressed, contrary to Emotionality and Extraversion, which are less pronounced. They are also characterized by moderate general self-efficacy. On the other hand, members of team sports produced the opposite results, as they are characterized by increased aggression, pronounced traits of Emotionality and Extraversion, somewhat less pronounced traits of Honesty-Humility, Openness to new experience, and less pronounced general self-efficacy. The percentage of explained variability of aggression is slightly higher in the subsample of team sports and constitutes 49.9% of the variability, while in the subsample of judokas it constitutes 47.8% of the variability of the criteria. Practical implications, limitations, and future research directions were discussed.

## Introduction

Judo is a Japanese martial art that is now widely recognized as an Olympic sport and it is the first martial art to be included in the Olympics in 1964. It is composed of the technical part, but also the moral and ethical part of the art (Fukuda et al., 2011). In 2012 Japan promoted martial arts (judo, kendo, and sumo wrestling) as a required subject in physical education. Schools have the possibility to select one or more martial arts, and to date, the majority of junior high schools have chosen judo (Yogi and Kyan, 2021). In addition, for 15 years the International Judo Federation together with National Federations, Ministries (Sports/Youth/Education), and National Olympic Committees have been conducting the Judo in Schools program. It is an extracurricular program widely supported across 49 countries. Also, an Erasmus + project named “SCHOOLJUDO.EU: A EU-Wide Primary School Ecosystem for the Judo Teaching” was started recently to promote the societal value of Judo, while advocating its standardization as part of the European primary teaching ecosystem (Erasmus + project ID 400623019). While team sports are highly appreciated and widely accepted in school curricula, with a documented positive impact on socio-psychological well-being (Zuckerman et al., 2021), the inclusion of judo raises many concerns even with the existence of scientific evidence that supports the relationship between martial arts practice and positive socio-psychological responses (Theeboom et al., 2009). Since judo incorporates fighting techniques, one of the main concerns is whether it can provoke aggressiveness and how it will influence the socio-psychological wellbeing of children and students. The data associating judo and/or martial arts with aggressiveness seem to be unclear.

At the youngest age (8 years), it seems that judo attracts children that scored higher on the anger scale compared to children who train karate or children who are not involved in any sports activity (Reynes and Lorant, 2001), and those results were even more pronounced after one (Reynes and Lorant, 2002) and 2 years of judo practice compared to other groups (Reynes and Lorant, 2004). The main support for the concerns was provided by Endresen and Olweus (2005), who claimed that participation in martial arts, boxing, wrestling, and weightlifting leads to an increase or enhancement of antisocial behavior in boys aged 11 to 13. They reported a large effect size value for the Violence Scale (above 1.0) in preadolescent and adolescent boys who had been continuously training combat sports over the total period of 2 years. Another finding suggests that secondary school students who engage in martial arts and combat sports show a higher approval of violence compared to non-athletes (Mutz, 2012). Still, the result of another study shows that martial arts and combat sports practitioners (aged 13 to 16) had a lower approval of violence compared to athletes who practice team sports, which are widely included in the school curriculum (Hortiguela et al., 2017). This study is missing a control group that would include non-athletes, so it was not possible to compare it with the previous one.

Somewhat indifferent results show that the aggressiveness of judokas does not differ from the group of non-athletes in boys (Ziaee et al., 2012) and girls (Lotfian et al., 2011) aged 11 to 19 (mean 12.90 ± 2.06 and 15.49 ± 1.93, respectively). A meta-analysis was conducted to review the relation between martial arts participation and externalizing behavior in juveniles (Gubbels et al., 2016). The conclusion was that levels of externalizing behavior do not differentiate martial artists from non-athletes and team sport athletes and that only athletes who practice individual sports had significantly better results.

The most recent studies gathered evidence about a positive influence of martial arts and combat sports on aggression (Harwood et al., 2017; Hortiguela et al., 2017; Wojdat et al., 2017; Coco et al., 2018; Kostorz and Sas-Nowosielski, 2021; Lafuente et al., 2021). Evidence that martial arts decrease aggression among the youth was found for nine intervention and longitudinal studies with a detected effect size of 0.65 (95% CI: 0.11, 1.03), which indicates a medium effect (Harwood et al., 2017). Although the results of systematic reviews and meta-analyses signal positive outcomes, all authors agreed that more research on the subject is necessary.

According to Salovey and Mayer (1990), emotional intelligence consists of abilities to perceive, assess, and express emotions quickly, recognize and generate the feelings that facilitate thinking, understand emotions and knowledge about emotions, and manage emotions to improve emotional and intellectual development. Emotional intelligence is treated as a trait or as an ability and, depending on the model, it can be measured using tests or estimated using self-assessment questionnaires (Petrides, 2011). Many studies indicate that more pronounced emotional intelligence is positively associated with sport participation in terms of success (Perlini and Halverson, 2006; Crombie et al., 2009; Laborde et al., 2016), satisfaction with sports performance (Laborde et al., 2014), experience of pleasant emotions (Lane and Wilson, 2011), and physical activity levels and positive attitudes toward physical activity (Laborde et al., 2016). Non-elite athletes with higher scores on managing emotions are more likely to use distraction-oriented coping. Compared to recreational athletes, judokas have more pronounced emotional intelligence (Acebes-Sánchez et al., 2021), especially managing emotions (Mitić et al., 2011).

However, the present study prioritizes the relationship between emotional intelligence and aggression. Lower scores for the trait emotional intelligence are connected with both physical and verbal aggression in adolescents of both genders (Inglés et al., 2021). Results of some studies (Bibi et al., 2020) suggest that emotional intelligence could be a protective factor against some aspects of aggression. Two systematic reviews claim that emotional intelligence and aggression are negatively related regardless of the ages, cultures, types of aggression (García-Sancho et al., 2014), and theoretical models of emotional intelligence (Vega et al., 2021). Both studies show that people with higher levels of emotional intelligence are less aggressive. However, regardless of these data, it should be noted that there are authors (Davis and Nichols, 2016) who (rightly) emphasize that “the more the better” does not seem to be right regarding emotional intelligence, and that there is an “optimal” level of emotional intelligence. For the present study, the fact that emotional intelligence can have a moderating role in the personality-aggression relationship (Peláez-Fernández et al., 2014) should be emphasized. The effectiveness of emotional intelligence-based learning interventions in reducing different types of aggression was established (Castillo et al., 2013). If it turns out that emotional intelligence is a strong predictor of reduced aggression, it is relevant for the practical application that some studies suggest that emotional intelligence can be developed through the practice of team sports (Campo et al., 2016), but also combat sports (Szabo and Urbán, 2014).

Self-efficacy can be defined as an individual’s assessment of his or her capacities to organize and execute specific actions necessary to achieve the desired goals (Bandura, 1999), and it represents a person’s confidence about goal achievement despite events, difficulties, or obstacles (Bandura, 1986). Self-efficacy can have an important role in sports success (Feltz et al., 2008) and can be improved in and through sport (Zagórska and Guszkowska, 2014). Self-efficacy, especially the social one, is associated with less aggressive behavior (Mofrad and Mehrabi, 2015). In adolescents, a significant and negative relationship between self-efficacy and overall aggression was determined, but there is a positive relationship between emotional self-efficacy and verbal aggression and hostility (Willemse et al., 2011). The relation between social self-efficacy and aggression is moderated by many different factors (Brubacher et al., 2016). In the sport context, the correlation of self-efficacy with aggressive behavior is negative in boxers, and self-efficacy was established as a significant negative predictor of aggressive behavior in boxers (Chen et al., 2019). The same authors claim that if there is a decrease in self-efficacy, the likelihood of aggressive behavior will increase.

The HEXACO model of basic personality structure (Lee and Ashton, 2008) was created during further studies of the dominant “Big five” model (McCrae and John, 1992; McCrae and Costa, 2008). It was found that, in addition to the five established personality traits (extraversion, neuroticism, openness, agreeableness, and conscientiousness), it would be necessary to include a sixth personality trait in the model. The sixth trait of personality has been confirmed and it is called “honesty/humility,” so the HEXACO model consists of Honesty, Emotionality, Extraversion, Agreeableness, Conscientiousness, and Openness (Ashton et al., 2004). Athletes from team sports have higher levels of extraversion and lower levels of conscientiousness in comparison to athletes participating in individual sports (Nia and Besharat, 2010; Allen et al., 2011). More successful athletes are characterized by a positive high score on emotionality and openness to experience (Mitić et al., 2021). By comparing champions of team sports and champions of individual sports (researched using the same methodology), it can be seen that all of them are characterized by a lower level of neuroticism and a higher level of extraversion. Still, champions of team sports are more open to experiences compared to less successful team sports athletes, and individual sports champions are characterized by a higher level of agreeableness and conscientiousness compared to less successful individual sports athletes (Piepiora, 2021a,b). Concerning the differences between athletes who practice combat sports and team sports, it was found that neuroticism and conscientiousness were the personality traits that distinguish the two groups (Bojanić et al., 2019). Dimension Honesty-Humility is most closely related to Negative Valence from BigFive + 2 (Međedović et al., 2019) and it has negative effects on aggression (Book et al., 2012; Dinić and Smederevac, 2019). The most important predictor of reactive aggression is Agreeableness (Dinić and Wertag, 2018). In the sport context, a statistically significant correlation was found between aggressiveness and extraversion, agreeableness, and emotional stability, and it was determined that emotional stability was a significant predictor of aggressiveness (Trninić et al., 2008).

There is some evidence that team sports athletes are more aggressive than athletes from individual sports (including MA and CS), but these findings came from the research that differs considerably in the methodology, sample characteristics, sports included, and outcomes (Ali et al., 2013; Mashhoodi et al., 2013). The present research aims to compare athletes who have trained judo for a prolonged time (minimum 5 years) and athletes from various team sports, primarily regarding the manifestation of aggression, but also regarding personality traits, emotional intelligence, and self-efficacy. Also, the aim was to examine the predictive value of personality traits, emotional intelligence, and self-efficacy for aggression within subsamples of judokas and team athletes. We hypothesize that there will not be a significant difference in aggressiveness, personality traits, emotional intelligence, and self-efficacy between judokas and team sports athletes (football, handball, and water polo). If confirmed, it would be an important addition to the open discussion.

## Materials and Methods

### Participants

The research sample consisted of 140 male respondents from Serbia (mean age 19.26), of whom 60 (42.9%) were judokas, while 80 (57.1%) were athletes who are active in team sports [football 34 (42.5%), handball 20 (25%), and water polo 26 (32.5%)]. At the time this research was conducted, all athletes included in the sample had been engaged in their chosen sport for at least 5 years, had at least 5 training sessions per week, and competed at least at the national level. Respondents completed (paper/pen) questionnaires at the scheduled time, 1 h before the start of the training session.

### Instruments

The instrument used to assess aggressiveness was Questionnaire A-87 (Žužul, 1989), which consists of 15 items of different situations with five possible responses. The possible responses or reactions are the five most frequent forms of aggressive responses: verbal manifest aggression (α = 0.84), physical manifest aggression (α = 0.89), indirect aggression (α = 0.90), verbal latent aggression (α = 0.89), and physical latent aggression (α = 0.93). The reliability of the questionnaire obtained in this study by calculating the Cronbach’s alpha is 0.970.

The instrument used to determine personality traits was HEXACO-PI-R (Lee and Ashton, 2006), which consists of 60 items in total. The dimensions represented were as follows: Honesty (α = 0.57), Emotionality (α = 0.74), Extraversion (α = 0.61), Agreeableness (α = 0.58), Conscientiousness (α = 0.71), and Openness (α = 0.59). The reliability of the questionnaire obtained in this study by calculating the Cronbach’s alpha is 0.756.

The emotional competence questionnaire (Takšić, 2002) consists of 15 statements where respondents give their answers by choosing one of the numbers given on a five-point scale. The answers represent the respondents’ assessment of the development of their abilities regarding emotional intelligence. In addition to the overall score, the scores using the subscales of Ability to perceive and understand emotions (α = 0.59), Ability to express and identify emotions (α = 0.70), and Ability to manage and regulate emotions (α = 0.68) were also obtained. The reliability of the questionnaire obtained in this study by calculating the Cronbach’s alpha is 0.770.

The instrument used to assess self-efficacy was the General Self-Efficacy Scale (GSE, Schwarzer and Jerusalem, 1995), which consists of 10 items, whereby respondents provide answers showing the extent to which the given statements are true to themselves, using a five-point Likert-type scale. The reliability of the questionnaire obtained in this study by calculating the Cronbach’s alpha is 0.837.

The Perceived Social Self-Efficacy (PSSE) Scale measures people’s beliefs in their capabilities to voice their own opinions with others, to work cooperatively and share personal experiences with others, and to manage interpersonal conflicts. PSSE is positively related to self-esteem, life satisfaction, and optimism (Caprara and Steca, 2005). The reliability of the questionnaire obtained in this study by calculating the Cronbach’s alpha is 0.914.

### Data Analysis

Descriptive measures, mean differences, hierarchical linear regression analysis, and discriminant analysis were used in the data analysis.

## Results

The research findings showed that professional judo athletes have statistically significant less pronounced indirect and physical manifest aggression compared to athletes from team sports (Table 2). Also, in relation to members of team sports, general self-efficacy is more pronounced among judokas and the obtained difference is statistically significant. In terms of personality traits, judokas have a significantly more pronounced Honesty-Humility trait and they are significantly more open to new experiences than those involved in team sports. On the other hand, judokas had a statistically significant lower level of Emotionality and Extraversion.

**TABLE 1:** Chi-square and functions at group centroids of canonical discriminant functions.

Wilks’ Lambda	Chi-square	Sig.	Function 1	
0.490	92.608	0.000	Judo	−1.167
			Team sports	0.876

**TABLE 2:** Means and structure matrix of canonical discriminant functions.

	Judo	Team sports	*F*	*p*	Stand. Can. Disc. Func. Coef.
	Mean	Mean			
Verbal manifest aggressiveness	34.6667	38.3250	3.231	0.074	0.271
Physical manifest aggressiveness	22.4333	26.6500	6.469*	0.012	–0.476
Indirect aggressiveness	19.7167	26.9625	19.405**	0.000	1.300
Verbal latent aggressiveness	31.1833	35.3875	3.944	0.049	–0.389
Physical latent aggressiveness	28.1833	31.2750	1.546	0.216	–0.037
Perceiving and understanding emotions	21.3333	21.7250	0.686	0.409	0.077
Expressing and identifying emotions	23.5333	24.4750	2.361	0.127	0.316
Managing and regulating emotions	14.9000	15.0000	0.065	0.799	0.253
General Self-Efficacy	35.2833	33.2625	8.395**	0.004	–0.351
Social self-efficacy	99.3833	98.1625	0.255	0.614	–0.028
Honesty-Humility	37.0667	34.7250	5.244*	0.024	–0.195
Emotionality	25.3000	27.8250	4.625*	0.033	0.367
Extraversion	32.2333	36.1625	18.346**	0.000	0.577
Agreeableness	31.3000	33.2125	3.800	0.053	0.630
Conscientiousness	38.0333	37.4375	0.360	0.549	0.086
Openness to experience	33.5833	29.8375	13.972**	0.000	–0.337

*Differences are significant at the 0.05 level and **differences are significant at the 0.01 level.

To check whether it is possible to distinguish judokas from athletes engaged in team sports, the method of canonical discriminant analysis was applied based on a set of variables that comprise aggression and its modalities, aspects of emotional competence, general and social self-efficacy, subjective assessment of general well-being, and personality traits according to the HEXACO model.

The values of the group centroids (average discriminant scores for each of the groups) range from −1.167 for judokas to 0.876 for athletes engaged in team sports (Table 1). The discriminant function was performed for sixteen factors, Wilks’ Lambda = 0.490. Chi-square = 92.608, *p* < 0.01.

Based on the data shown in Table 2, it can be concluded that judokas are characterized by a low degree of aggression, especially low indirect and physical manifestations of aggression. In addition, the personality traits Honesty-Humility and Openness to experience are well expressed, contrary to Emotionality and Extraversion, which are less pronounced. They are also characterized by moderate general self-efficacy. On the other hand, members of team sports produced the opposite results, as they are characterized by increased aggression, pronounced traits of Emotionality and Extraversion, somewhat less pronounced traits of Honesty-Humility, Openness to new experience, and less pronounced general self-efficacy.

Given that a significant number of predictor variables are present in the study, it was also examined to what extent it is possible to predict the severity of aggression and its models based on a set of predictor variables consisting of personality traits according to the HEXACO model, aspects of emotional competence—expression, recognition, and control of emotion, and general and social self-efficacy.

Prediction models were shown to be statistically significant, both on the judo sample and on the team sports sample (Table 3). The percentage of explained variability of the criterion variable aggression is slightly higher in the subsample of team sports athletes and constitutes 49.9% of the variability, while in the subsample of judoka it constitutes 47.8% of the variability of the criteria.

**TABLE 3:** Hierarchical linear regression analysis.

Model	R	R Square	df	F	Sig.
Subsample: Judo	0.692	0.478	59	4.003	0.000
Subsample: Team sports	0.706	0.499	79	6.149	0.000

In the subsample of team athletes, a significant partial contribution to the explanation of the variability of the criterion variable aggression is made by the variables Agreeableness (*t* = −3.868, *p* < 0.01), Extraversion (*t* = −3.772, *p* < 0.01), and Emotionality (*t* = −2.784, *p* < 0.01). Beta coefficients are negative, which indicates that the expression of these personality traits is inversely proportional to the expression of aggression in team athletes.

In the subsample of judokas, a statistically significant partial contribution to the explanation of the variability of the criterion variable aggression is made by the variables Agreeableness (*t* = −3.125, *p* < 0.01) and Conscientiousness (*t* = 2.339, *p* < 0.05), which means that in this subsample a higher degree of Agreeableness leads to a lower expression of aggression (negative beta coefficient) while a higher expression of Conscientiousness leads to higher aggressiveness (positive beta coefficient).

## Discussion and Conclusion

The results obtained in this study show that judokas display statistically significant lower overall aggressiveness compared to team sports athletes, as well as significantly lower expressed indirect and physical manifest aggression. This result is in line with previous research reporting a positive impact of martial arts on prosocial behaviors (Harwood et al., 2017; Hortiguela et al., 2017; Wojdat et al., 2017; Coco et al., 2018; Kostorz and Sas-Nowosielski, 2021; Lafuente et al., 2021). The obtained results not only justify the inclusion of judo in school curricula but also give it a certain advantage over more popular and widely accepted team sports. The data relating to significantly less pronounced physical aggression in judokas are particularly interesting and significant since they are in direct contrast to the prejudices related to martial arts, which were formed based on external observation of these sports.

This study also examined whether it is possible to distinguish judokas from athletes who play team sports based on a set of variables that comprise aggression and its modalities, aspects of emotional intelligence, general and social self-efficacy, and personality traits. Judokas, unlike team sports athletes, are characterized by more pronounced personality traits Honesty-Humility and Openness to Experiences and less pronounced traits of Emotionality and Extraversion, as well as more pronounced general self-efficacy and less pronounced physical manifest aggressiveness and indirect aggressiveness.

The more pronounced general self-efficacy of judokas can be explained by the fact that it is an individual sport, in which it is easier to develop confidence in one’s value and work on achieving goals, regardless of any possible obstacles. Athletes in individual sports must rely exclusively on themselves, i.e., their abilities, while participants in team sports share the credit for both success and failure. This thesis is supported by the fact that a statistically significant difference was obtained only with regard to general, but not social, self-efficacy. This finding should be viewed in the light of previous research, which suggests that self-efficacy in martial arts—specifically in boxing—is not only important for sports performance but also has a protective role in preventing physical and mental problems (Chen et al., 2019). In terms of personality traits, judokas are significantly more open to new experiences and have a more pronounced Honesty-Humility dimension compared to those involved in team sports. This result can be at least partially and logically explained by the requirements of the sport in which the athletes are engaged. A high score on the Honesty-Humility dimension also implies manipulating others for personal gain, which is less characteristic (or necessary) for judo compared to team sports. A higher level of Openness to experience is characteristic of risky sport participants (Tok, 2011), which judo certainly is when compared to team sports. Furthermore, judokas had a statistically significant lower level of Emotionality and Extraversion. Achieving lower scores on the Emotionality scale characterizes, among other things, people who are not afraid of the possibility of physical injury and do not need to share their worries with others (Ashton et al., 2004), which are characteristics more suitable for training and competition in judo than in team sports. Also, this result is in line with the results of previous studies (Bojanić et al., 2019). Extraversion, on the other hand, has subordinating aspects: social self-confidence, social courage, sociability, and activity (Ashton et al., 2004), which are, by their nature, related to collective activities.

The research also examined whether and to what extent it is possible to predict the severity of aggression based on a set of predictor variables that comprise personality traits according to the HEXACO model, aspects of emotional competence, and general and social self-efficacy in judokas and team athletes. Interestingly, in both subsamples, the prediction models proved to be significant and explained approximately the same percentage of variance (49.9 and 47.8%) of the criterion aggression. What both models have in common is that the personality trait Agreeableness has a significant partial contribution to explaining the aggressiveness with which it is inversely proportional. This is in line with earlier findings (Dinić and Wertag, 2018). Persons with very high scores on the Agreeableness scale are characterized by the fact that they will forgive the injustices they have suffered, judge others mildly, be willing to compromise and cooperate, and be able to easily control their nature (Ashton et al., 2004), so this finding was expected. In the subsample of team athletes, it was found that Extraversion and Emotionality, in addition to Agreeableness, are also significant predictors of aggression. Expression of these personality traits is inversely proportional to the expression of aggression. The obtained results are in agreement with the results of an earlier study (Trninić et al., 2008), in which the majority of the total sample (109 athletes) played ball sports (76) and only 15 practiced combat sports. In the subsample of judokas, in addition to the dimension of Agreeableness, Conscientiousness is also a significant predictor of aggression in such a way that more pronounced Conscientiousness leads to higher aggression.

What is surprising is the fact that emotional intelligence and generalized and social self-efficacy are not significant predictors, although this was expected based on previous research reporting an association between emotional intelligence and lower aggressiveness (García-Sancho et al., 2014; Vega et al., 2021) and significant negative predictor roles of self-efficacy in aggressiveness in boxers (Chen et al., 2019). Future research should focus on the moderating role of emotional intelligence in the personality-aggression relationship, as suggested by some authors (Peláez-Fernández et al., 2014), since the sample size was limited in this study. Regarding self-efficacy, it is necessary to find sport-specific moderators who influence the relationship between social self-efficacy and aggression, as suggested in previous studies (Brubacher et al., 2016).

The proposed suggestions for further research are directly related to the limitations of this study. These limitations primarily appertain to the sample, which is not large enough to perform analyses such as moderator effects. In addition, the sample included only male participants so it would be beneficial that future research includes females. Also, it must be stated that the sport success of athletes involved in this research was not controlled, and the objectives of the research cannot be achieved on subsamples defined on the basis of success, which would certainly be significant. Furthermore, the questionnaires used in this research are not sports-specific. The reason is that the basic thread for such research was the importance and expediency of including martial arts (specifically judo) in school curricula and that the possible general psychosocial effects, rather than sports success, were the primary value.

Despite these limitations, it is safe to consider the objectives of the research as achieved. Less pronounced aggression with all its subdomains in judokas compared to athletes who play team sports certainly breaks the prejudices related to martial arts that many people have due to the similarity of numerous techniques with manifestations of aggression outside sports facilities. The obtained results not only equate judo with team sports in terms of inclusion in the school curriculum but also favor it in a certain way. The obtained predictors of aggression in athletes who engage in different types of sports can help coaches and sports psychologists to better understand the aggressive behavior of athletes, but also to prevent it more effectively.

## Data Availability Statement

The raw data supporting the conclusions of this article will be made available by the authors, without undue reservation.

## Ethics Statement

The studies involving human participants were reviewed and approved by Ethics Committee of Faculty of Sport and Physical Education, University of Nis. The patients/participants provided their written informed consent to participate in this study.

## Author Contributions

NemS, DT, NM, MM, and NenS designed the experiments, analyzed and interpreted the data, edited the manuscript, and approved the final version to be published and are accountable for all aspects of the work. NemS and DT performed the experiments and wrote the initial draft of the manuscript. All authors contributed to the article and approved the submitted version.

## Conflict of Interest

The authors declare that the research was conducted in the absence of any commercial or financial relationships that could be construed as a potential conflict of interest.

## Publisher’s Note

All claims expressed in this article are solely those of the authors and do not necessarily represent those of their affiliated organizations, or those of the publisher, the editors and the reviewers. Any product that may be evaluated in this article, or claim that may be made by its manufacturer, is not guaranteed or endorsed by the publisher.
